# Decoupling Solar Variability and Instrument Trends Using the Multiple Same-Irradiance-Level (MuSIL) Analysis Technique

**DOI:** 10.1007/s11207-018-1294-5

**Published:** 2018-04-23

**Authors:** Thomas N. Woods, Francis G. Eparvier, Jerald Harder, Martin Snow

**Affiliations:** Laboratory for Atmospheric and Space Physics, University of Colorado, 3665 Discovery Drive, CO 80303 Boulder, USA

**Keywords:** Solar spectral irradiance, Solar cycle variability, Ultraviolet-visible-infrared radiation

## Abstract

The solar spectral irradiance (SSI) dataset is a key record for studying and understanding the energetics and radiation balance in Earth’s environment. Understanding the long-term variations of the SSI over timescales of the 11-year solar activity cycle and longer is critical for many Sun–Earth research topics. Satellite measurements of the SSI have been made since the 1970s, most of them in the ultraviolet, but recently also in the visible and near-infrared. A limiting factor for the accuracy of previous solar variability results is the uncertainties for the instrument degradation corrections, which need fairly large corrections relative to the amount of solar cycle variability at some wavelengths. The primary objective of this investigation has been to separate out solar cycle variability and any residual uncorrected instrumental trends in the SSI measurements from the *Solar Radiation and Climate Experiment* (SORCE) mission and the *Thermosphere, Mesosphere, Ionosphere, Energetic, and Dynamics* (TIMED) mission. A new technique called the Multiple Same-Irradiance-Level (MuSIL) analysis has been developed, which examines an SSI time series at different levels of solar activity to provide long-term trends in an SSI record, and the most common result is a downward trend that most likely stems from uncorrected instrument degradation. This technique has been applied to each wavelength in the SSI records from SORCE (2003 – present) and TIMED (2002 – present) to provide new solar cycle variability results between 27 nm and 1600 nm with a resolution of about 1 nm at most wavelengths. This technique, which was validated with the highly accurate total solar irradiance (TSI) record, has an estimated relative uncertainty of about 5% of the measured solar cycle variability. The MuSIL results are further validated with the comparison of the new solar cycle variability results from different solar cycles.

## Introduction

The solar spectral irradiance (SSI) dataset is a key record for studying and understanding the energetics and radiation balance in Earth’s environment. Understanding the long-term variations of the SSI over timescales of the 11-year solar activity cycle and longer is critical for many Sun–Earth research topics. Global-scale studies sometimes only need the total solar irradiance (TSI, solar irradiance integrated over all wavelengths) measurements, but regional studies and detailed modeling of the atmosphere, surface, and ocean energetics require the solar spectral irradiance (SSI). The following are examples of Sun–Earth studies that can benefit from a more accurate SSI record over many decades.

### Radiative Energy Balance

The TSI and SSI climate records are important for constraining the complicated energy flows within the climate system and for resolving disputed energy estimates (Stephens *et al.*, 2012; Wild *et al.*, 2013, 2015; L’Ecuyer *et al.*, 2015).

### Solar Heating Bottom Up

Solar variations, volcanic activity, the El Niño Southern Oscillation (ENSO), and anthropogenic gases are the primary forcings that alter climate (*e.g.* Mann *et al.*, 2005; Lean and Rind, 2008). Global warming is mainly due to increasing anthropogenic gases, and solar irradiance variability is estimated to cause about 10% of the 0.74 C per century increase in global surface temperature (Lean and Rind, 2008).

### Solar Heating Top Down

Significant uncertainty and controversy remain in model simulations for the solar ultraviolet photochemistry of ozone and heating in the stratosphere and then dynamically coupling to below (Haigh *et al.*, 2010; Garcia, 2010; Merkel *et al.*, 2011; Swartz *et al.*, 2012; Matthes *et al.*, 2016). Improved understanding of SSI variability is required for these studies.

### Solar Cycle Variability

The amount and phasing of the solar cycle variability as a function of wavelength between 300 nm and 1000 nm has been controversial since the *Solar Radiation and Climate Experiment* (SORCE) SSI results were published by Harder *et al.* (2009). Many of the differences between the SORCE and other measurements and solar models have not yet been resolved (Ball *et al.*, 2011; Unruh, Ball, and Krivova, 2012; DeLand and Cebula, 2012; Ermolli *et al.*, 2013; Marchenko and DeLand, 2014; Woods *et al.*, 2015). More analyses such as discussed here and as expected with the new Total and Spectral Irradiance Sensors (TSIS) SSI observations are anticipated to address this issue.

### Long-Term Solar Variability and Climate Change During the Maunder Minimum

The satellite-era SSI observations are critical in helping to reconstruct solar variability during the Maunder Minimum (*e.g.* Lean, 2000), and these estimates are used to model possible climate change during this period of very low solar activity (*e.g.* Rind *et al.*, 2004; Feulner, 2011; Ineson *et al.*, 2015; Maycock *et al.*, 2015). Studies of the Maunder Minimum period provide a range of possibilities for how important solar forcings can be for climate change and are becoming ever more relevant as the solar activity has declined in Solar Cycle 24 (2009 – present) and is predicted to decline even more for Solar Cycle 25.

To support these and other Sun–Earth studies, several satellite measurements of the SSI have been made since the 1970s that contribute to understanding the solar variability over Solar Cycles (SC) 21 to 24. Most of these SSI measurements have been made in the ultraviolet, but recently also in the visible and near-infrared. Combining these different observations into an accurate composite SSI record has two primary challenges: 1) removing instrument trends to obtain precise solar cycle trends, and 2) establishing a reference scale for the irradiance at each wavelength. This second challenge has been largely addressed with an extensive validation effort during the UARS mission (Woods *et al.*, 1996) and also through the publication of accurate solar reference spectra, such as by Woods *et al.* (2009), Harder *et al.* (2010), and Thuillier *et al.* (2004, 2014). The first challenge is an ongoing issue, however, and has limited the accuracy of the current understanding of solar cycle variability and thus the development of more accurate SSI variability models. This new Multiple Same-Irradiance-Level (*MuSIL*) technique directly addresses this challenge for the SSI measurements from SORCE and *Thermosphere, Mesosphere, Ionosphere, Energetic, and Dynamics* (TIMED).

Understanding the 11-year solar cycle variations in the ultraviolet at wavelengths shorter than 208 nm (Al ionization edge) is an easier problem because the amount of solar variability is 20% to even a factor of 10 at the shortest wavelengths. Even instruments with a stability capability of 2% per year can accurately determine the solar cycle variability for these wavelengths. For wavelengths at longer than 208 nm, the solar cycle variability is as low as 0.05% at many wavelengths in the visible and infrared ranges, so that instruments with stability of 0.005% (50 ppm) per year are needed. Meeting a stability requirement is reduced to understanding instrument degradation trends to better than this stability requirement. This can be challenging for this stability requirement, and understanding instrument degradation is at the heart of the controversy for solar cycle variations from the SORCE *Spectral Irradiance Monitor* (SIM) (Harder *et al.*, 2009). These SORCE SIM measurements indicate that there is more near-ultraviolet (NUV: 300 – 400 nm) solar cycle variation than earlier observations reported (Harder *et al.*, 2009; Unruh, Ball, and Krivova, 2012; DeLand and Cebula, 2012; Ermolli *et al.*, 2013). These variations have also been debated on the basis that they are inconsistent with some SSI model estimates (*e.g.* Ball *et al.*, 2011; Pagaran *et al.*, 2011). Furthermore, some out-of-phase solar cycle variation for various visible (VIS: 400 – 800 nm) and near-infrared (NIR: 800 – 1600 nm) wavelengths reported by Harder *et al.* (2009) are not reproduced in these particular model studies. The SORCE SIM degradation trends are based primarily on weekly measurements with a redundant calibration channel (SIM B) and modeling the degradation as a function of exposure time for both the daily channel (SIM A) and SIM B. A key concern for understanding the solar cycle variability measurements from SORCE SIM is how much non-exposure degradation may exist for its low-duty-cycle calibration channel. The MuSIL method explores how much uncorrected instrument degradation could be contained in the SORCE data trends.

**Figure 1:**
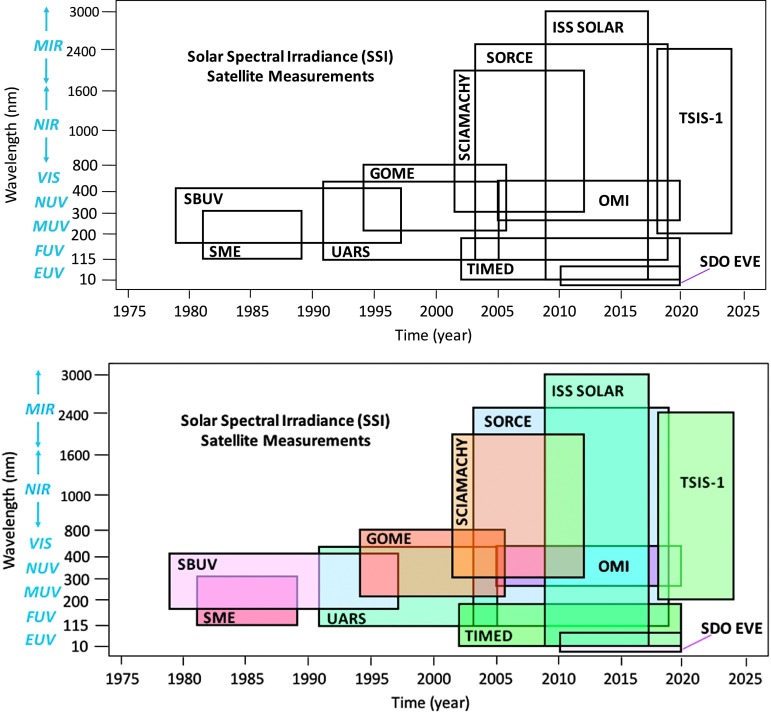
Graphic display of the SSI satellite measurements as a function of wavelength and time. The MuSIL analysis technique has been applied to the SORCE and TIMED SSI data, and future efforts will provide results from other missions.

Many solar ultraviolet (UV) spectral irradiance observations have been made since the 1970s, as illustrated in Figure 1 and listed in Table 1. The satellite-era of solar UV irradiances began with the *Nimbus-7 Solar Backscatter UltraViolet* (SBUV) (Schlesinger and Cebula, 1992) in the 160 to 400 nm range and continued with NOAA-9 SBUV/2 (DeLand, Cebula, and Hilsenrath, 2004) and NOAA-11 SBUV/2 (DeLand and Cebula, 1998). NASA solar UV irradiance measurements over similar UV wavelengths include the *Solar Mesospheric Explorer* (SME) (Rottman, 1988), the *Upper Atmosphere Research Satellite* (UARS) *Solar Ultraviolet Spectral Irradiance Monitor* (SUSIM) (Brueckner *et al.*, 1993), the UARS *Solar Stellar Intercomparison Experiment* (SOLSTICE) (Rottman, Woods, and Sparn, 1993), and the SORCE *Solar Stellar Irradiance Comparison Experiment* (SOLSTICE) (McClintock, Rottman, and Woods, 2005). These SSI measurements are made over the far-ultraviolet (FUV: 115 – 200 nm), mid-ultraviolet (MUV: 200 – 300 nm), and NUV (300 – 400 nm). DeLand and Cebula (2008) have developed a composite of the solar UV irradiance using measurements obtained between 1978 and 2005.

**Table 1:** Solar Spectral Irradiance (SSI) Instruments and Calibration Methods. The Pt/Ne/Cr lamps only provide a wavelength calibration for GOME and SCIAMACHY.

Mission/instrument and mission years	Wavelength range/resolution (nm)	Calibration methods		
		Redundant channels	On-board source	External calibration
SDO/EVE 2010 – present	6 – 106 nm/0.1 nm	Multiple filters	LED lamps	Calibration rockets
SOLAR/SOLSPEC 2008 – 2017	180 – 3000 nm/1 – 8 nm	No	Tungsten & D2 lamps	–
SOLAR/SolACES 2008 – 2017	17 – 220 nm/0.3 – 0.9 nm	Multiple filters	Ion cell	–
OMI 2004 – present	265 – 500 nm/0.5 nm	Multiple diffusers	–	–
SORCE/SOLSTICE 2003 – present	115 – 308 nm/0.1 nm	Yes	–	Stars
		A & B		
SORCE/SIM 2003 – present	240 – 2400 nm/1 – 10 nm	Yes	–	–
		A & B		
SCIAMACHY 2002 – 2012	230 – 1750 nm/1 nm	No	Pt/Ne/Cr lamp	–
TIMED/SEE-EGS 2002 – present	27 – 190 nm/0.4 nm	Yes	Hg lamp	Calibration rockets
		1 Cal Ch.		
GOME 1994 – 2006	240 – 790 nm/0.3 nm	No	Pt/Ne/Cr lamp	–
UARS/SOLSTICE 1991 – 2005	117 – 420 nm/0.2 nm	No	–	Stars
UARS/SUSIM 1991 – 2005	115 – 410 nm/0.1 – 1 nm	Multiple optics & sensors	D2 lamps & Tungsten lamps	–
SME 1981 – 1989	115 – 300 nm/1 nm	No	–	Calibration rockets
NOAA SBUV 1-2 1978 – 1997	160 – 400 nm/1 nm	No	–	–

The SSI measurements were extended in wavelength coverage in the 2000s. The extension over the extreme ultraviolet (EUV: 10 – 115 nm) is made possible by the TIMED *Solar Extreme ultraviolet Spectrometer* (SEE) *EUV Grating Spectrograph* (EGS) (Woods *et al.*, 2005), the *Solar Dynamics Observatory* (SDO) *Extreme ultraviolet Variability Experiment* (EVE) (Woods *et al.*, 2012), and the *Solar Auto-Calibrating EUV Spectrometers* (SolACES) (Schmidtke *et al.*, 2014). NASA’s expansion into the visible (VIS: 400 – 800 nm) and NIR is made possible by the SORCE *Spectral Irradiance Monitor* (SIM) (Harder *et al.*, 2005). Three SSI datasets are also available in the VIS and NIR ranges from the European Space Agency (ESA): the *Global Ozone Monitoring Experiment* (GOME) (Burrows *et al.*, 1999), the *Scanning Imaging Absorption Spectrometer for Atmospheric CHartographY* (SCIAMACHY) (Pagaran, Weber, and Burrows, 2009), and the *Solar Spectrum* (SOLSPEC) (Thuillier *et al.*, 2009). The German SolACES and French SOLSPEC instruments are integrated onto the ESA SOLAR platform that was deployed onto the *International Space Station* (ISS).

The future SSI observations in the NUV-VIS-NIR ranges will be obtained with the next generation of the SIM instrument onboard the *Total Spectral Irradiance Sensor* (TSIS-1) mission to the ISS and in the *Compact Spectral Irradiance Monitor* (CSIM) CubeSat. Both of these SSI measurements are expected to start in 2018. While no future missions are planned for full spectral coverage in the EUV or FUV, key EUV and FUV bands are available for the operational *EUV X-ray Irradiance Sensor* (EXIS) (Eparvier *et al.*, 2009) onboard the NOAA GOES-R series of four satellites; the first in the series (R) was launched in November 2016 and was renamed GOES-16.

Several SSI models have been validated with some of these SSI measurements. The models also provide SSI estimates for use in Sun–Earth studies, but because they are not measurements, they are only briefly mentioned here. The three primary SSI models that have been used for atmosphere and climate modeling are the NRLSSI (Lean *et al.*, 1997, 2005), SATIRE (Fligge, Solanki, and Unruh, 2000; Ball *et al.*, 2014), and SRPM (Fontenla *et al.*, 1999, 2011, 2014). The SATIRE model uses magnetograms for solar variability and was initially developed with the UARS SUSIM measurements to improve the SATIRE model in the 115 – 180 nm range. The latest SATIRE-S model uses SORCE SOLSTICE data for its FUV range (Yeo *et al.*, 2014). The NRLSSI and SRPM models used UARS SSI data for their development, and their more recent versions use SORCE SSI data. The latest version of NRLSSI-2 has been adopted by NOAA for its SSI Climate Data Record (Coddington *et al.*, 2016, 2017).

The following sections provide an overview of in-flight calibration methods and their potential shortcomings, an introduction of the new MuSIL analysis technique to uncouple solar cycle variability and any residual uncorrected instrumental trends, and the MuSIL analysis results for the TIMED SEE, SORCE SOLSTICE, and SORCE SIM data. The final sections discuss the comparison of new solar cycle variability results and future plans to perform the MuSIL analysis on other previous SSI datasets.

## Instrument In-Flight Calibration Methods

This research investigates the SSI data trends to decouple solar variability and instrument degradation trends, although it does not directly address the cause of the instrument degradation. Nonetheless, a short description of the instrument in-flight calibration methods is presented to illustrate the challenges with instrument calibrations and to describe why trends may still remain in a dataset even after robust in-flight calibration corrections have been included in the data processing.

A variety of methods have been developed for tracking the degradation of an instrument during its mission, and each method has its advantages, but also some limitations. Table 1 provides a list of the SSI instruments that will be used in the proposed research, and their in-flight calibration methods.

The most common in-flight calibration method, as first demonstrated well with TSI instruments, is using redundant channels such that one channel is used daily and other channels are used less frequently, such as once per week or once per month. The primary assumption for this method is that degradation is related directly to exposure time, and so the least-exposed channel will have no (or at least very little) degradation during the mission and serves as reference to correct all other channels. The key limitation with the redundant-channel calibration method is that no direct measure of the degradation of the least-exposed channel is available, and so systematic degradation, such as by energetic particles in the space environment that degrade the onboard optics, sensors, or electronics, might be affecting all channels; thus trending of the least-exposed channel can be difficult to understand accurately.

Another type of in-flight calibration method is the use of onboard calibration lamps. The assumption for this method is that the source is stable and can provide in-flight calibrations of the sensors. There are four known limitations for onboard lamps. One is that they usually only provide degradation tracking of the sensors, but not of the optics, if the lamp shines only on the sensors. Another limitation is that the lamp illumination on the optics and sensor usually does not match the solar field of view; thus there can be systematic offsets for the lamp degradation trend that differ from the solar-illumination degradation trend. The third limitation is that the lamps themselves can degrade, and this concern can be partially mitigated by having multiple, redundant sources on board, as is done by UARS SUSIM. A fourth limitation is that the lamps can fail during flight, such as was the case for the SOLSPEC $\mbox{D}_{2}$ calibration lamps.

Another form of onboard calibrations is gain calibrations for the sensor electronics. An onboard reference voltage or current can be used to calibrate the electronics gain in order to track how sensor electronics might be changing over a mission. One limitation is that this type of calibration does not provide a calibration of the sensor itself. Another limitation is that the reference electronics source can also degrade over the mission.

A third in-flight calibration technique is to use external calibrations. The early version of this technique was the use of underflight calibration experiments, which are flown about once a year so that their fresh calibration can be transferred to the satellite instrument. This technique has been used for SME, SBUV, TIMED SEE, and SDO EVE. The primary limitation of this technique is that the transfer of the calibration is limited to the accuracy of the underflight instrument calibration, and this accuracy is often higher than the stability requirement for the satellite instrument. For example, the accuracy of the pre-flight calibrations for SORCE SOLSTICE is about 2% using the best possible radiometric standard for the ultraviolet (UV: 120 – 400 nm), being the NIST Synchrotron Ultraviolet Radiation Facility (SURF). However, this 2% accuracy is higher than its stability requirement of 0.5% per year, so underflight calibration experiments for SOLSTICE can be helpful on a timescale of four years, but are not as useful for a one- to two-year timescale. This limitation was realized for the SME and SBUV underflight experiments after the small amount of solar variability in the longer wavelengths was better understood and the stability requirements were revised to lower values; this was the motivation for designing SOLSTICE instruments to use bright, stable (main-sequence) stars for their external calibrations instead of underflight calibrations. The wavelength ranges that are still effective for using underflight calibration experiments are the EUV and FUV because the solar cycle variability in these ranges is about a factor of two, and thus they have less stringent stability requirements of 2% per year or greater, which are thus comparable to the SURF calibration accuracy of ${\sim}\,2\%$.

Both UARS SOLSTICE and SORCE SOLSTICE use bright, stable stars for their in-flight calibration to track instrument degradation. Some limitations exist for the stellar calibrations for these two missions, however. One limitation is that the SOLSTICE optics are illuminated differently for the solar and stellar observations, and this limitation is largely mitigated with monthly cruciform scans over the field of view to measure the exposure-related burn-in near the center of the optics. The other limitation is that stellar calibrations were not made after the UARS and SORCE satellites both had power issues, and thus stellar calibrations ended several years before the mission end. The MuSIL analysis can address these concerns.

The solar irradiance community has learned from these limitations of in-flight calibrations over the decades, and improvements continue to be made. For example, the TSIS SIM instrument has numerous improvements, such as having two redundant calibration channels instead of one, as was the case for SORCE SIM; therefore, the TSIS solar observations will be a benchmark for solar irradiance observations and for improved in-flight calibrations for better understanding any instrument trends. The motivation for developing a new method for data analysis is to use the lessons learned and reanalyze previous SSI observations to better decouple the true solar cycle variability and instrument degradation trends. Section 3 discusses this new MuSIL technique for improving previous SSI datasets.

## Decouple Instrument Degradation with the MuSIL Analysis Technique

The newly developed MuSIL analysis technique can be used to separate out solar cycle variability and any residual uncorrected instrumental trends in an SSI long-term dataset. This section introduces the MuSIL analysis technique, describes the new estimates for uncorrected instrument trends that the technique provides, and reports the validation of the MuSIL solar cycle variability results after it was applied to the SORCE and TIMED SEE datasets.

The initial motivation for developing a new technique to decouple solar cycle variability and instrument degradation trends came from working with the TIMED SEE solar EUV-FUV dataset, because its underflight calibration program ended in 2008 and a new need arose to track its degradation differently. The SEE redundant channel that is used weekly for calibration, SDO EVE calibration experiments in the EUV, and overlap in the FUV with SORCE SOLSTICE have helped to track TIMED SEE degradation trends since 2008, but residual trends remained in the TIMED SEE version 11 data products that were suggestive of uncorrected instrument degradation *versus* true solar cycle variability. In particular, the concern is that low solar values from TIMED SEE in 2016 are lower than the solar cycle minimum values in 2008 at some wavelengths. The new technique that has helped to understand the TIMED SEE trends is referred to as the MuSIL analysis technique.

The basis for the MuSIL technique is the assumption that there are times before and after solar cycle minimum (or maximum) at which the irradiance is at the same level. If these times can be identified using accurate irradiance data, then a non-flat trend between these times for the dataset being analyzed could indicate uncorrected instrument degradation. Because different wavelengths can peak at different times during the 27-day solar rotation period, it is important to perform this analysis with at least 27-day smoothing of the irradiance time series. A single level of solar activity limits the time range for such an analysis, so that this approach is extended to cover the full range of a mission by combining analyses for multiple levels of solar activity. The approach for the MuSIL analysis is to first establish a reference “super proxy” that is considered reliable for selecting the times for the same irradiance levels, then combining the trends from multiple levels, and finally fitting trend functions over the full mission.

**Figure 2:**
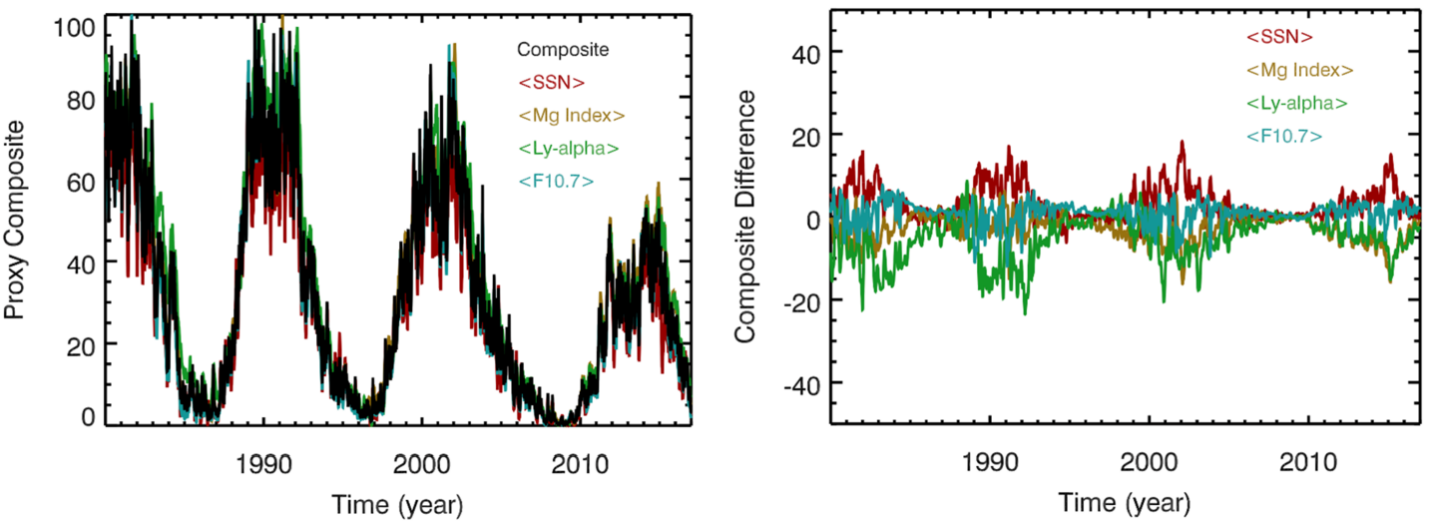
Proxy composite, or “super proxy”: average of the sunspot number (SSN, photosphere), Mg ii index (chromosphere), H i Lyman-alpha (transition region), and F10.7 (corona). Each proxy is normalized to be between 0 and 100 before the average is made. The differences between each of these proxies to the proxy composite are shown in the right panel.

The super proxy developed for the MuSIL analysis uses accurate composite time series of emissions appropriate to be proxies of solar variability in the photosphere, chromosphere, transition region, and corona layers of the solar atmosphere. The chosen proxies for these solar layers are the sunspot number (Clette *et al.*, 2014), the Mg ii (280 nm) core-to-wing ratio index (Viereck *et al.*, 2004), H i (121.6 nm) Lyman-alpha (Woods *et al.*, 2000; Woods and Rottman, 1997), and the 10.7 cm radio flux (F10.7) (Tapping, 2013). Each of these four solar variability proxies are normalized to be between 0 and 100 (cycle minimum to maximum), then averaged together to be the super proxy composite, also in the range of 0 to 100. These four normalized proxies and the resulting proxy composite are shown in Figure 2 with 27-day smoothing, along with a plot of the differences between each proxy and the super proxy. These differences indicate that there is a small systematic negative offset of the Mg index and Ly-alpha proxies relative to the super proxy, and that there is a small positive offset of the SSN and F10.7 relative to the super proxy. The average standard deviation of these differences is 3.8 for the four different proxies; this value is one indication (validation) that the result for the MuSIL technique uncertainty could be at least 4% of the solar cycle variability. These differences between the super proxy and the individual proxies are expected to be representative of the differences between the super proxy and the SSI emissions that are also from the photosphere, chromosphere, transition region, and corona. These four proxies are in phase with each other over the solar cycle, so that any irradiance time-series comparisons to the super proxy that yield a negative correlation would represent variability that is out of phase with the solar cycle.

It is noted that the different proxies have larger differences during solar cycle maximum, but that the transitions between high and low solar cycle levels (declining phase) and between low and high (rising phase) are more consistent between the different proxies. Therefore, the MuSIL analysis approach has the potential to be more accurate by performing the analysis during the transition periods. For the MuSIL analysis of the TIMED and SORCE data shown here, the selected activity levels range from 5 to 45 in increments of 5, whereby the times of the same irradiance levels are selected based on the 27-day smoothing of the super proxy. That is, nine different solar activity levels are used for the MuSIL analysis, and these levels avoid using solar maximum levels, except for Solar Cycle 24. Because of the requirement to have measurements before and after solar cycle minimum (or maximum), this MuSIL technique is more effective for missions of eight years or longer.

Because there are differences in center-to-limb variation for the solar radiation at different wavelengths, the super proxy and the irradiance dataset are smoothed over 27 days (solar rotation period) to enable a more accurate selection of the times for the same irradiance level. For the 15-year long TIMED SEE mission and the 14-year long SORCE mission, there are typically 10 to 20 different 27-day intervals that have at least one day within 2% of a specified super proxy level. The day within each of these 27-day intervals with the smallest difference from the reference level is selected for the MuSIL trend analysis of the irradiance record. The first level analyzed is level 30, and a linear fit over time is derived for the resulting irradiance trend. Then the other levels are analyzed, and their linear fits of the irradiance trends are scaled to match the magnitude of the reference (first) level-30 trend. In particular, the scale factor for the extra irradiance trend at a different super proxy level is the average of the ratio of the extra trend linear fit to the reference (first) trend linear fit. These ratios are calculated only for the time period that overlaps with both the extra trend range and reference (first) trend range; that is, extrapolation in time is not allowed for the scale factor calculation. When all level time points are combined in this way, the trend over the full mission is fit.

**Figure 3:**
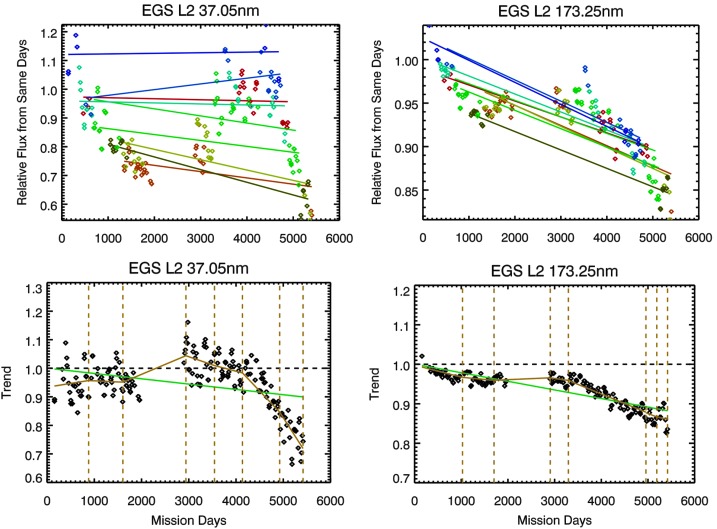
Example MuSIL analysis and trend fits for TIMED SEE data at 37.05 nm and 173.25 nm. The top plots show the trends for each irradiance level (each has a different color) that has both solar cycle variability and instrument degradation. The *lines* in the top plots are linear fits per level, which ideally would be flat lines if there were no instrument degradation. The bottom plots show these data merged into a single instrument degradation trend. The *green line* in the bottom plot is a single linear fit over the full mission. The MuSIL result is the mission-long fit as piecewise linear fits (*gold lines*).

**Figure 4:**
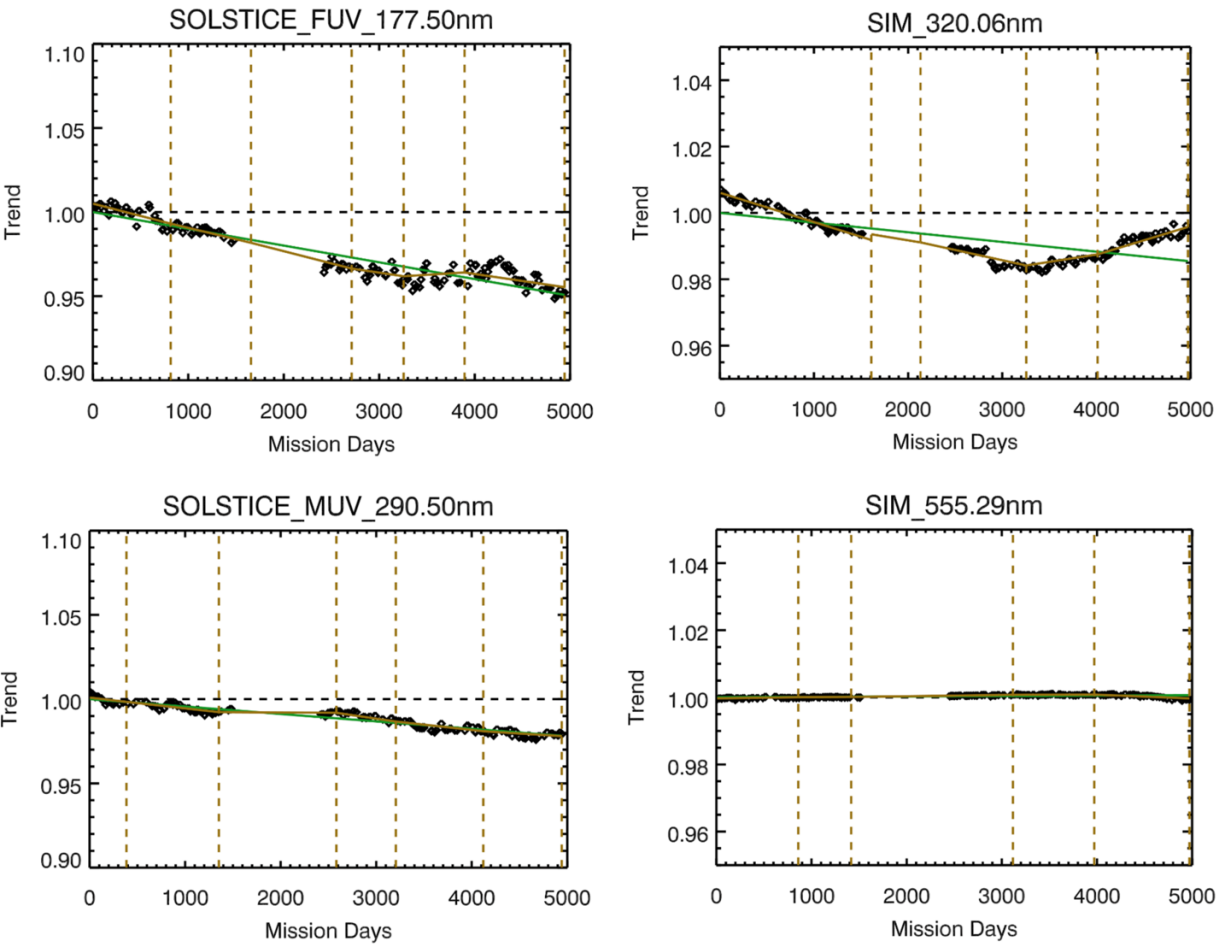
Example trend fits for SORCE SOLSTICE data at 177.5 nm and 290.5 nm and for SORCE SIM at 320.06 nm and 555.29 nm. These results use the same MuSIL analysis technique as is illustrated in Figure 3. All SORCE trends are lower than 0.4% per year.

An example of these trends is shown in Figure 3 for two wavelengths in the TIMED SEE data. The individual level trend fits are similar but not accurate by themselves because there are only a few data points per level. The merged set is much more accurate for trending and has more complete coverage over time, except for the time gap at solar cycle minimum. Because the gap during solar cycle minimum is inherent in the MuSIL approach, the resulting trend fits are likely less accurate during minima. All known corrections have already been applied for the TIMED SEE data, so any trend is considered to be uncorrected instrument degradation. This version of the TIMED SEE data only had degradation corrections applied to 2011, which means that declines after TIMED mission day 3300 were expected to be found. The mission-long fit can be any function; for now, a piecewise linear fit every two years appears to be a good method for the TIMED SEE and SORCE SSI datasets. The vertical dashed lines in the bottom plots of Figure 3 are the boundaries between the piecewise fits; these boundaries are initially on two-year intervals and then adjusted automatically as part of the fit procedure so that the fits are continuous (no jumps). Examples of the MuSIL analysis and mission-long piecewise fits are also shown for some wavelengths in Figure 4 for SORCE SOLSTICE and SIM.

It is important to note that the MuSIL technique is not a proxy model development, but an analysis to extract long-term trends. The resulting long-term trend could be related to instrument degradation that has not been applied yet or could be solar cycle variability that is different than the super proxy cycle variability. It is easy to tell the difference between these two options: if the resulting trend is monotonically decreasing (or increasing), then the trend is mostly related to instrument degradation (or recovery), and if the resulting trend has an 11-year cycle that is in phase or out of phase with the solar cycle, then the trend is mostly related to an inappropriate proxy used in the analysis. In the MuSIL analysis of 1800 different wavelengths in the TIMED SEE and SORCE SSI datasets, only the case for instrument degradation has been found; of course, a very small solar cycle variation may remain in the MuSIL resulting trends that the initial analysis approach did not detect.

**Figure 5:**
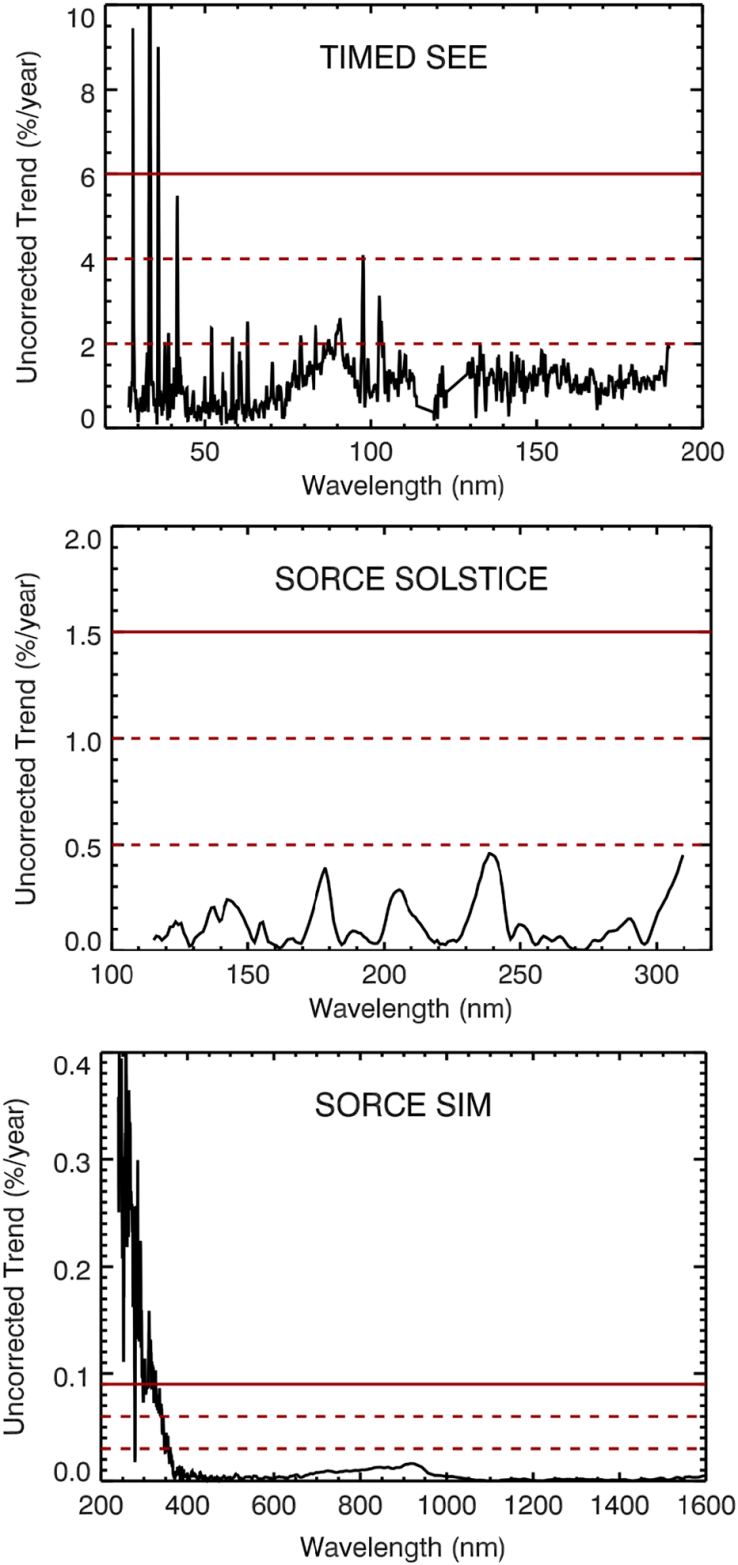
Average rate for uncorrected degradation trend for the TIMED and SORCE instruments. The estimated stability uncertainty for each instrument’s public data product is provided as the *red dashed lines* for 1-sigma and 2-sigma and as a *red solid line* for 3-sigma.

The amount of uncorrected degradation found in the MuSIL analysis during the TIMED and SORCE mission is mostly within 1-sigma of the stability uncertainty estimated for each dataset as based on calibration corrections made in data processing. Figure 5 shows the mission-average rate of uncorrected degradation found with the MuSIL analysis technique, along with the estimated stability for the TIMED and SORCE data products. For TIMED SEE, the stability uncertainty is about 2% per year and is limited by its measurement precision for the weekly redundant (low-duty-cycle) channel calibration. The stability uncertainty for SORCE SOLSTICE is improved to 0.5% per year and is limited by the trending analysis of stellar measurements at a few wavelengths. The SORCE SIM stability uncertainty is improved even more to 0.03% per year and is limited by its measurement precision for the monthly redundant (low-duty-cycle) channel calibration. That the MuSIL results are generally lower than the instrument stability uncertainties indicates that the instrument original datasets are perhaps as accurate as possible for their in-flight degradation tracking techniques. This also means that the application of the MuSIL results is a small correction relative to the instrument stability uncertainty, but the MuSIL results can make significant differences for a solar cycle variability analysis for wavelengths where the solar cycle variability is smaller than the instrument stability uncertainty.

In some wavelengths, the uncorrected degradation trend from the MuSIL result is larger than the 3-sigma instrument stability estimate. For TIMED SEE, these higher trend rates lie between 27 and 40 nm. These wavelengths have a solar cycle variability of a factor of about three, which translates into a significant intrinsic solar variability over a week; the weekly redundant calibrations for TIMED SEE EGS for these wavelengths may not be frequent enough. The SDO EVE redundant filter calibrations are improved with a daily calibration with secondary filters and a weekly calibration with tertiary filters. The SORCE SOLSTICE results are within its 1-sigma stability estimate for all wavelengths. The SORCE SIM results are within its 3-sigma stability estimate, except for shorter than 327 nm. The SORCE SIM results at these shorter wavelengths are likely limited to the measurement precision for these UV wavelengths for the weekly redundant calibrations. A possible conclusion is that the redundant SIM calibration channel is degrading more than expected in the ultraviolet. These predicted uncorrected degradation trends might be useful to reevaluate the in-flight calibration corrections and those techniques, but finding a root cause for the small amount of the uncorrected degradation trend may not be feasible. The spectral overlap in the 200 – 300 nm range by SOLSTICE and SIM helps to provide validation for the MuSIL results when the revised SOLSTICE and SIM solar cycle variability results agree (as discussed in the next section).

## Validation of the MuSIL Analysis Technique

These MuSIL resulting trends are then applied to the original irradiance time series to provide a revised time series that is intended to represent a fully corrected dataset. By design, the MuSIL technique is expected to force the relative spectral variability from different solar cycles from a single instrument to be the same. In addition, the true solar cycle variability during a specific solar cycle should be the same from different instruments. Therefore, any differences in solar cycle variability from different instruments and from different solar cycles can provide an estimate of the uncertainty of the MuSIL technique.

The solar irradiances selected for the solar cycle variability comparisons are 27-day averages centered on 2003 July 18 for the Solar Cycle 23 (SC 23) maximum, on 2009 January 01 for solar cycle minimum, and on 2012 June 2 for the SC 24 maximum. The solar variability is calculated as the maximum irradiance divided by the minimum irradiance and then minus unity. That is, the variability is the relative amount of irradiance change above the cycle minimum irradiance. The solar cycle maxima dates were chosen based on the same-irradiance-level for the super proxy to be slightly below the SC 24 true maximum. Because the SC 24 variability is much reduced, by a factor of two at some wavelengths, compared to the SC 23 variability, the solar cycle variability results presented here are about a factor of two lower than the SC 23 full variability.

**Figure 6:**
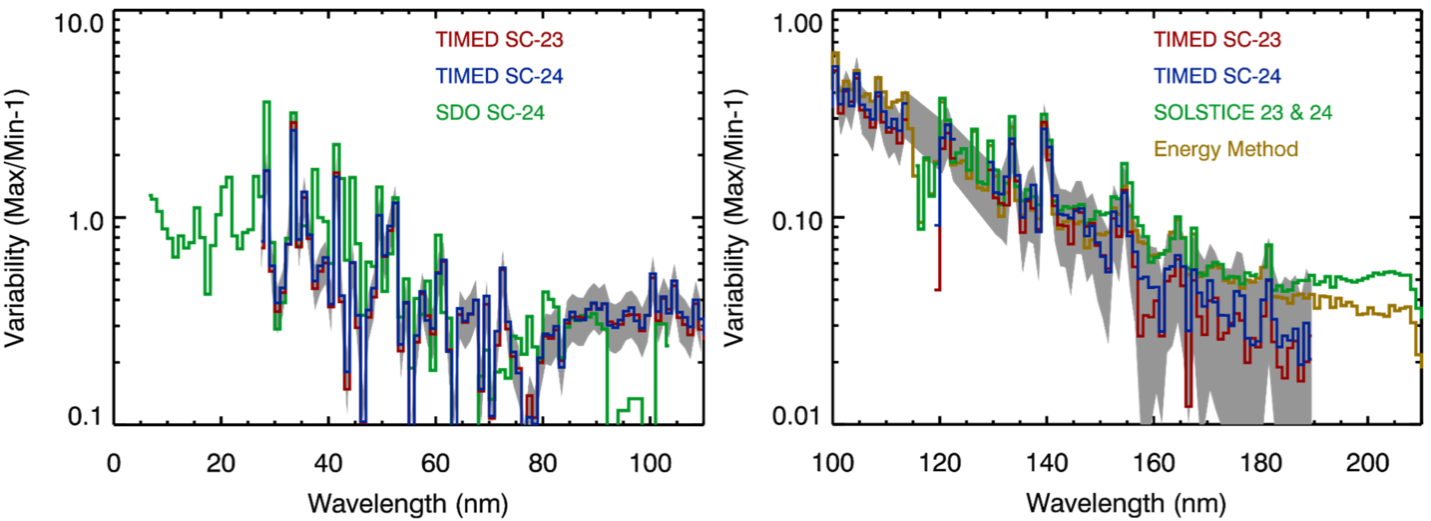
TIMED SEE EGS solar cycle variability comparisons after the MuSIL analysis results have been applied. The consistency of the Solar Cycle 23 (SC-23, *red*) and 24 (*blue*) variabilities validates the MuSIL technique. The *gray shading* is the uncertainty for applying the MuSIL analysis results. The energy method model for solar cycle variability (Woods *et al.*, 2015) has a similar spectral variability as these results with the MuSIL analysis. The SDO variability result has not been analyzed with MuSIL so far, but the SOLSTICE result was analyzed with MuSIL.

**Figure 7:**
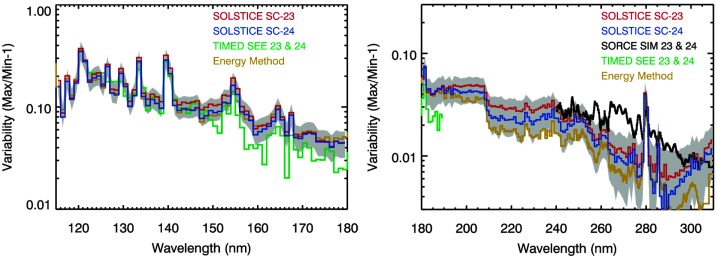
SORCE SOLSTICE solar cycle variability comparisons after MuSIL analysis results have been applied. The consistency of the Solar Cycle 23 (SC-23, *red*) and 24 (*blue*) variabilities validates the MuSIL technique. The *gray shading* is the uncertainty for applying the MuSIL analysis results. The energy method model for solar cycle variability (Woods *et al.*, 2015) has a similar spectral variability as these results with the MuSIL analysis. The TIMED SEE and SORCE SIM results have also been analyzed with MuSIL.

**Figure 8:**
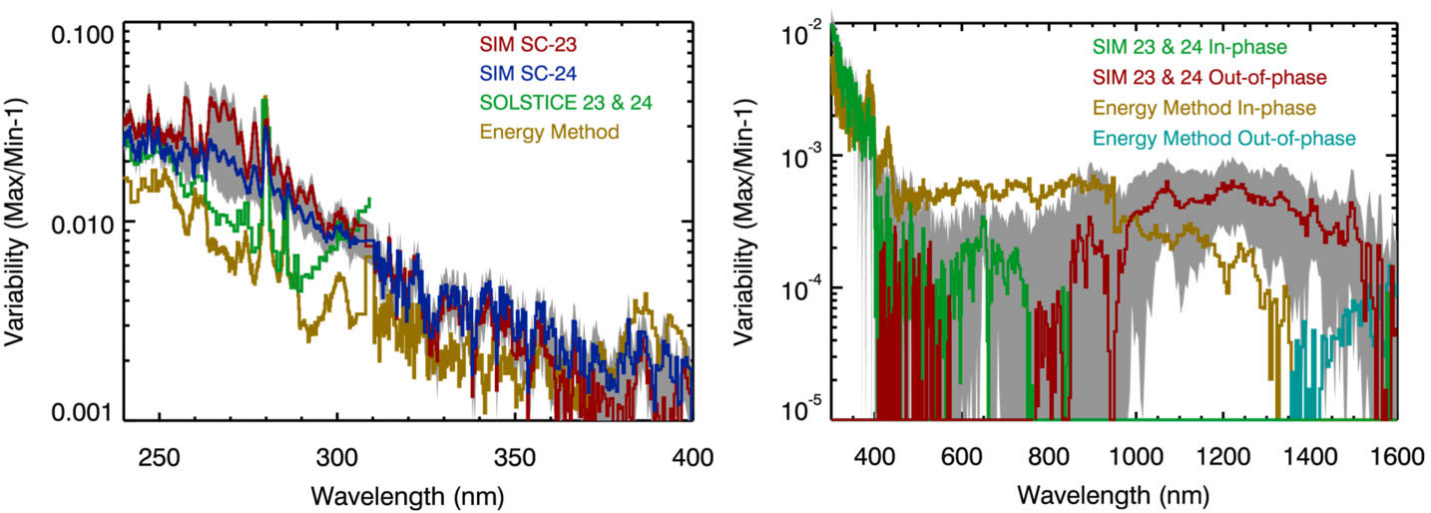
SORCE SIM solar cycle variability comparisons after MuSIL analysis results have been applied. The consistency of the Solar Cycle 23 (SC-23, *red*) and 24 (*blue*) variabilities in the left plot validates the MuSIL technique. The *gray shading* is the uncertainty for applying the MuSIL analysis results. The energy method model for solar cycle variability (Woods *et al.*, 2015) has a similar spectral variability in the MUV and NUV (240 – 400 nm) as these results with the MuSIL analysis, but the differences in the VIS and NIR (400 – 1600 nm) are larger, in part because there is less solar cycle variability at longer than 400 nm.

Figures 6, 7, 8 show the solar cycle variability after the MuSIL analysis corrections were applied for the TIMED SEE and SORCE SSI datasets, respectively. Based on these comparisons, the uncertainty of the MuSIL analysis results is estimated as the difference between two different solar cycles. This uncertainty, shown as the gray shaded area in Figures 6 – 8, is about ${\pm}\,10\%$ of the solar cycle variability.

**Figure 9:**
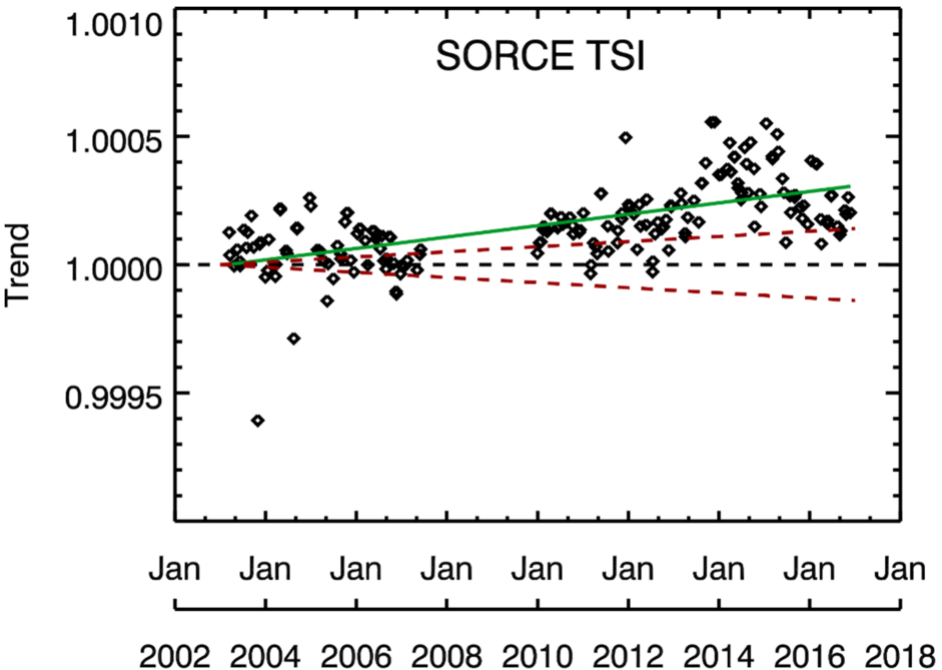
MuSIL analysis for the SORCE TIM. The *green line* is a linear fit of the MuSIL results over the full SORCE mission. The *red dashed lines* represent the TIM stability uncertainty of 10 ppm per year; this corresponds to 140 ppm (0.014%) in 2017. The MuSIL result is within 2-sigma of the TIM stability trend. When the TSI data are considered to be perfect, then this indicates that the MuSIL technique uncertainty is about 4% of the solar cycle variability.

As another validation approach for the MuSIL analysis technique, the very accurate TSI record from the SORCE *Total Irradiance Monitor* (TIM) is analyzed. It is assumed that any deviations from a flat trend for the SORCE TSI result could represent the uncertainty of the MuSIL technique. This analysis is shown in Figure 9. There is a slight trend for the TSI analysis of 20 ppm per year, and this trend is about a factor of two larger than the TIM TSI stability uncertainty of 10 ppm per year. If one assumes that the SORCE TSI time series is a perfect dataset, then this MuSIL trend result suggests that the MuSIL technique uncertainty is about 4% of the solar cycle variability.

An alternative interpretation is that there is an uncorrected trend in the SORCE TSI data. The MuSIL trend result for the SORCE TSI does not show a solar cycle variation, which would indicate TSI differences between the super proxy; instead, the MuSIL trend result shows a linear trend over the SORCE mission, which could indicate an uncorrected trend for the SORCE TSI data. Dewitte and Nevens (2016) reported an increase in SORCE TSI trend relative to their TSI composite of 25 ppm per year, which is slightly steeper than the MuSIL result (green line) shown in Figure 9. Are these SORCE TSI trends from the MuSIL analysis and from the Dewitte and Nevens (2016) analysis an indication of non-exposure-related recovery (enhanced sensitivity) for the low-duty-cycle radiometers in SORCE TIM? Additional studies of the TSI measurements from the French *Picard* mission, the Air Force/NOAA/NASA *TSI Calibration Transfer Experiment* (TCTE) mission, and the future more accurate TSI measurements from TSIS could help answer this question. Nonetheless, this estimated relative uncertainty from the MuSIL analysis of the SORCE TSI data is consistent with the uncertainty estimates for the SSI solar cycle variability comparisons after the MuSIL results were applied to the SSI data.

A potential concern with using the super proxy for the TSI analysis, and also for SSI wavelengths at longer than 300 nm, is that the super proxy does not have a component for the dark sunspot deficit contribution to TSI. The sunspot deficit effect for the TSI (and for many SIM SSI wavelengths) is greatly reduced using 27-day smoothed time series for the MuSIL analysis because dark sunspots typically affect the TSI for only a few days. Furthermore, an analysis of the daily TSI and Lyman-alpha variability over the many 27-day solar rotation periods during the SORCE mission indicates that the TSI and Lyman-alpha are in phase with each other over a solar rotation 80% of the time and only 20% of the time out of phase. Again, 27-day smoothing of the time series before performing the MuSIL analysis greatly reduces any daily or solar rotation differences between the super proxy and an irradiance time series.

Based on these MuSIL analyses for SORCE and TIMED SSI data, a conclusion is that the MuSIL technique is most effective if 1) the SSI dataset has daily observations over eight years or longer in order to cover declining and rising phases of solar cycles, 2) the best possible calibrations and trend corrections have been applied to the SSI dataset before the MuSIL analysis is performed, and 3) the measurement precision is much better than the amount of intrinsic solar cycle variability. The other datasets appropriate for this analysis include the SSI data from OMI, UARS, SME, and SBUV. The ESA SSI datasets do not meet all of these conditions and thus are not appropriate for analysis with the MuSIL technique. These excluded SSI datasets are the ISS SOLSPEC because it does not have daily observations, the SCIAMACHY because no calibration corrections are applied to account for icing on the cooled detectors and the sudden changes in calibration after detector bakeouts, and the GOME because no calibration corrections are applied and its solar data are not publicly available. It is feasible to consider a MuSIL analysis of the SCIAMACY and GOME solar data when improved datasets are available.

The MuSIL technique appears to work very well for UV wavelengths shorter than 400 nm, but the uncertainties of the MuSIL analysis results for the VIS and NIR ranges are not much better than those for the energy method solar cycle results from Woods *et al.* (2015). The VIS and NIR ranges are challenging for the MuSIL technique because there is so little solar cycle variability. An additional analysis with the SORCE data might improve the MuSIL technique, and improved trend fitting results for the VIS and NIR ranges may then be possible.

## Comparison of Solar Cycle Variability

A composite result of the new estimates of solar cycle variability is made for comparison to models of SSI variability. This composite used the MuSIL-based results from TIMED SEE for 27 nm to 115 nm, from SORCE SOLSTICE for 115 nm to 300 nm, and from SORCE SIM for 300 nm to 1600 nm. As mentioned in Section 4, the data selected for the solar cycle variability comparisons are 27-day averages centered on 2003 July 18 for the SC 23 maximum, on 2009 January 01 for solar cycle minimum, and on 2012 June 2 for the SC 24 maximum.

**Figure 10:**
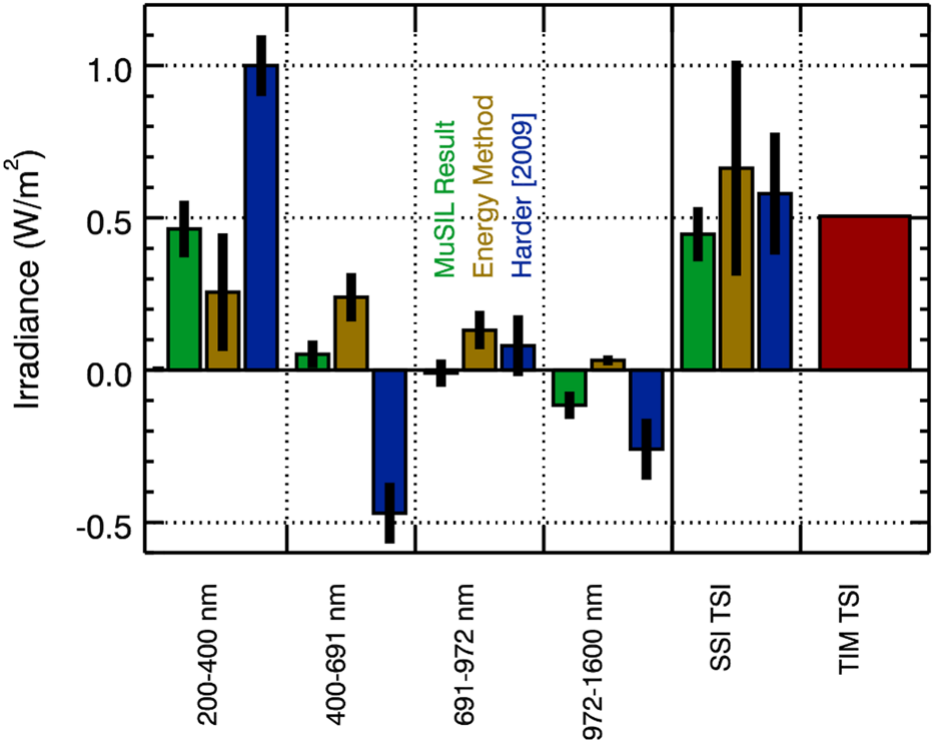
Solar cycle variability comparison between the MuSIL results (*green*), the energy method model (*gold*), and results from Harder *et al.* (2009) (*blue*). All three integrated over wavelength agree with the TSI variability measured by SORCE TIM, but they disagree significantly among themselves in the different bands.

These MuSIL results are compared over broad bands in Figure 10 to the energy method model (Woods *et al.*, 2015) and SORCE direct results given by Harder *et al.* (2009). In this plot, the variability is the cycle maximum minus cycle minimum and thus is in units of $\mbox{W/m}^{2}$. The integration of this variability over wavelength (115 – 1600 nm) for these three different SSI analyses provides a very similar solar cycle variability irradiance of about $0.5~\mbox{W/m}^{2}$, which is in agreement with the SORCE TIM measurement of the TSI cycle variability. These three estimates are in reasonable agreement for the 691 – 1600 nm range, but the Harder *et al.* (2009) results have much larger differences than the other two results for the 200 – 691 nm range. The MuSIL and energy method results used data from both SC 23 and 24, whereas the Harder *et al.* (2009) result is obtained during the declining phase of SC 23 alone. The MuSIL analysis suggests that there are uncorrected instrument degradation trends in the SORCE SIM data and that the amount of uncorrected degradation is larger at wavelengths shorter than 400 nm. With the relative uncertainty of the energy method being about 30% of the solar cycle variability, the MuSIL results are considered more accurate than the energy method results.

**Figure 11:**
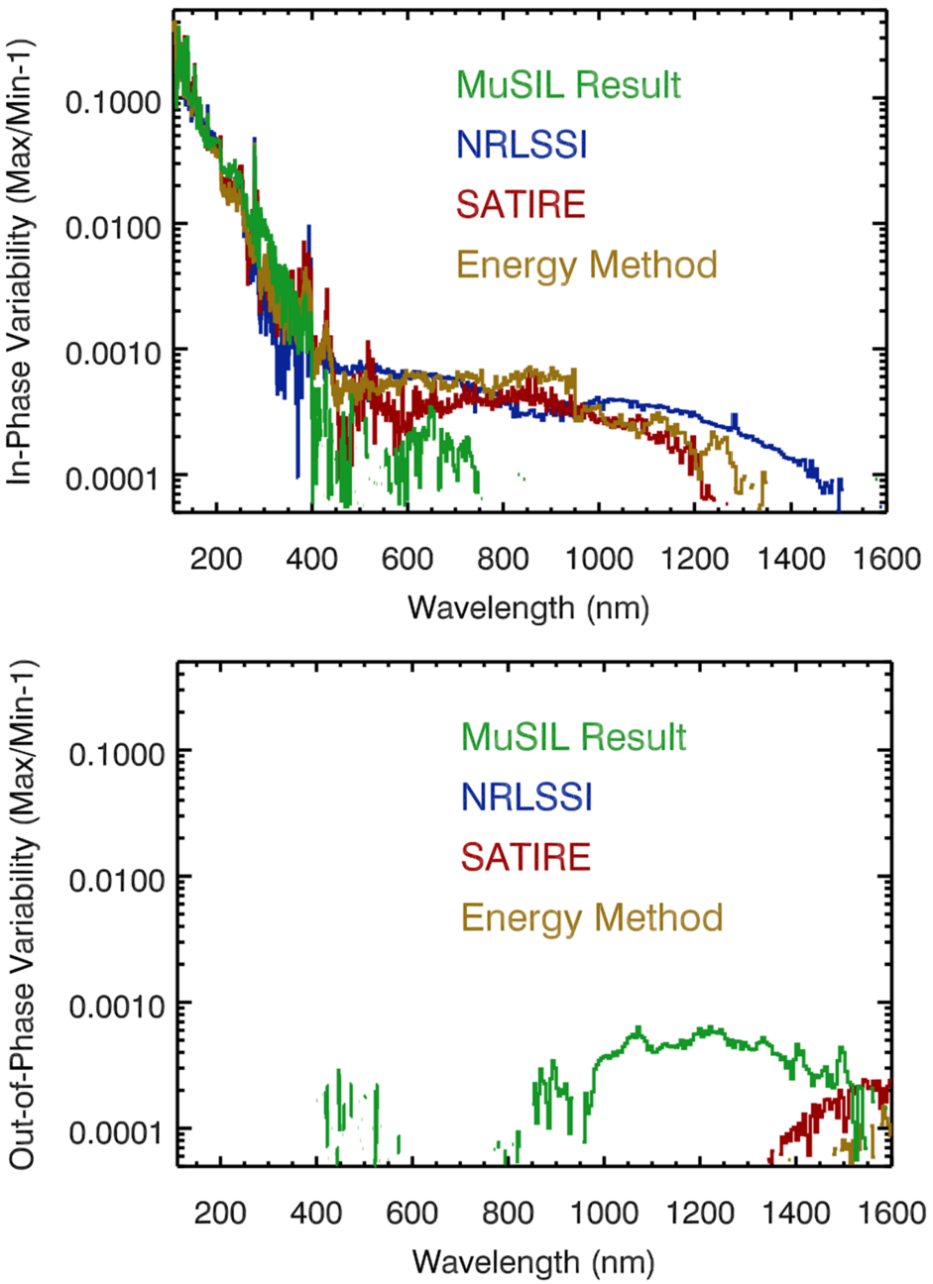
Solar cycle variability comparison between the MuSIL results (*green*), SATIRE-S model (*red*), NRLSSI-2 model (*blue*), and the energy method model (*gold*). There is similarity at wavelengths shorter than 700 nm, but there are significant differences from the MuSIL result in the 700 – 1500 nm range.

These MuSIL results are also compared at higher spectral resolution to the SATIRE-S and NRLSSI-2 model estimates of solar cycle variability, as shown in Figure 11. There is similarity at wavelengths shorter than 700 nm, but there are significant differences for the MuSIL results in the 700 – 1500 nm range. The MuSIL result is the only one in this figure that shows out-of-phase variability between 800 and 1300 nm; the others estimate in-phase variability for that range. The SATIRE-S model agrees best with the MuSIL solar cycle variability result for wavelengths shorter than 700 nm and also at wavelengths longer than 1400 nm. The MuSIL results are perhaps more similar to the SCIAMACHY solar variability model estimate for the SC 23 variability, which does show out-of-phase variability for 800 to 1700 nm (Pagaran, Weber, and Burrows, 2009). The SCIAMACHY solar data do not yet have robust calibration corrections applied because of icing on the cooled detectors, but for their SCIAMACHY solar variability analysis, Pagaran, Weber, and Burrows (2009) performed piecewise sunspot/facula modeling over data sections in between the detector bakeouts. If the new, more accurate TSIS SSI observations confirm these MuSIL results, then updates for the SSI variability models will be appropriate.

## Summary and Future Work

The MuSIL analysis technique provides new estimates for uncorrected instrument degradation trends for a long-term solar irradiance time series. These results applied to the original data then provide new estimates for solar cycle variability. The relative uncertainty for these new variability results is about 10% of the solar cycle variability. For example, at a wavelength with 1% solar cycle variability, the uncertainty is 0.1% for the new variability. The new results of solar cycle variability were presented from 27 nm to 190 nm using TIMED SEE data and were presented from 115 nm to 1600 nm using SORCE SOLSTICE and SIM data. There is consistency in the spectral variability between 115 nm and 400 nm for these new MuSIL-based results using TIMED SEE and SORCE SOLSTICE data and the energy method model (Woods *et al.*, 2015). Differences occur in the solar cycle variability at longer than 400 nm, however. These differences are only about 0.05%, but this is large compared to the solar cycle variability of about 0.07% or smaller in the VIS-NIR ranges. Of particular interest is which wavelengths are out of phase with the solar cycle. The MuSIL-based results for the SORCE SIM data indicate in-phase variability at shorter than 700 nm and out-of-phase variability between 700 and 1600 nm. The MuSIL results between 400 nm and 1600 nm have differences between the SORCE SIM results by Harder *et al.* (2009), the energy method model, and other solar SSI variability models (*e.g.* SATIRE or NRLSSI). The largest difference in the VIS-NIR ranges is between the SORCE SIM Harder *et al.* (2009) results in the 400 – 700 nm range, which indicates out-of-phase variability, and the MuSIL results and all other models, which indicate in-phase variability. It may not be possible to resolve these differences in the VIS-NIR ranges until more accurate/precise measurements are available, such as those anticipated from the TSIS SIM, starting in 2018.

The MuSIL results for uncorrected instrument degradation were integrated into the TIMED SEE data processing, and its version 12 data products will have improved solar EUV-FUV irradiance results. This is the primary outcome intended from the original motivation to develop the new MuSIL analysis technique. The MuSIL results for the SORCE SSI and TSI data will be examined more carefully, and there may be some new insights for the SOLSTICE, SIM, and TIM trends that might lead to additional degradation corrections in their future data products.

Consistency of analysis for the different SSI records will help to make a new multi-instrument SSI composite record, so that other previous SSI datasets will be analyzed with this new MuSIL analysis technique. A new SSI composite record from the 1970s to the present and with wavelength coverage from 6 nm to 3000 nm will be a valuable resource for Earth scientists who are studying and modeling Earth’s radiation budget, solar heating effects in the atmosphere and at the surface, and any other Sun–climate data analysis and modeling efforts. This improved SSI record can then be compared to the new TSIS measurements, and these comparisons will help to further validate this new MuSIL technique with the accurate TSIS observations and to extend this new composite SSI record into the future.

## References

[CR1] Ball W.T., Unruh Y.C., Krivova N.A., Solanki S., Harder J.W. (2011). Solar irradiance variability: A six-year comparison between SORCE observations and the SATIRE model. Astron. Astrophys..

[CR2] Ball W.T., Krivova N.A., Unruh Y.C., Haigh J.D., Solanki S.K. (2014). A new SATIRE-S spectral solar irradiance reconstruction for Solar Cycles 21 – 23 and its implications for stratospheric ozone. J. Atmos. Sci..

[CR3] Brueckner G.E., Edlow K.L., Floyd L.E., Lean J.L., VanHoosier M.E. (1993). The Solar Ultraviolet Spectral Irradiance Monitor (SUSIM) experiment on board the Upper Atmosphere Research Satellite (UARS). J. Geophys. Res..

[CR4] Burrows J.P., Weber M., Buchwitz M., Rozanov V., Ladstätter-Weißenmayer A., Richter A., Debeek R., Hoogen R., Bramstedt K., Eichmann K., Eisinger M., Perner D. (1999). The Global Ozone Monitoring Experiment (GOME): Mission concept and first scientific results. J. Atmos. Sci..

[CR5] Clette F., Svalgaard L., Vaquero J.M., Cliver E.W. (2014). Revisiting the sunspot number. A 400-year perspective on the solar cycle. Space Sci. Rev..

[CR6] Coddington O., Lean J.L., Pilewskie P., Snow M., Lindholm D. (2016). A solar irradiance climate data record. Bull. Am. Meteorol. Soc..

[CR7] Coddington O., Lean J., Pilewskie P., Woods T. (2017). Newly released climate data records of total and spectral solar irradiance are based on SORCE observations. GSICS Q. Newsl..

[CR8] DeLand M.T., Cebula R.P. (1998). NOAA 11 Solar Backscattered Ultraviolet, model 2 (SBUV/2) instrument solar spectral irradiance measurements in 1989 – 1994. 2: Results, validation, and comparisons. J. Geophys. Res..

[CR9] DeLand M.T., Cebula R.P. (2008). Creation of a composite solar ultraviolet irradiance data set. J. Geophys. Res..

[CR10] DeLand M.T., Cebula R.P. (2012). Solar UV variations during the decline of cycle 23. J. Atmos. Solar-Terr. Phys..

[CR11] DeLand M.T., Cebula R.P., Hilsenrath E. (2004). Observations of solar spectral irradiance change during cycle 22 from NOAA-9 Solar Backscattered Ultraviolet Model 2 (SBUV/2). J. Geophys. Res..

[CR12] Dewitte S., Nevens S. (2016). The total solar irradiance climate data record. Astrophys. J..

[CR13] Eparvier F.G., Jones A.R., Chamberlin P.C., Woods T.N., McClintock W.E., Snow M. (2009). The Extreme UltraViolet Sensor (EUVS) for GOES-R. Proc. SPIE.

[CR14] Ermolli I., Matthes K., Dudok de Wit T., Krivova N.A., Tourpali K., Weber M., Unruh Y.C., Gray L., Langematz U., Pilewskie P., Rozanov E., Schmutz W., Shapiro A., Solanki S.K., Woods T.N. (2013). Recent variability of the solar spectral irradiance and its impact on climate modelling. Atmos. Chem. Phys..

[CR15] Feulner G. (2011). Are the most recent estimates for Maunder Minimum solar irradiance in agreement with temperature reconstructions?. Geophys. Res. Lett..

[CR16] Fligge M., Solanki S.K., Unruh Y.C. (2000). Modelling irradiance variations from the surface distribution of the solar magnetic field. Astron. Astrophys..

[CR17] Fontenla J., White O.R., Fox P.A., Avrett E.H., Kuruca R.L. (1999). Calculation of solar irradiances. I. Synthesis of the solar spectrum. Astrophys. J..

[CR18] Fontenla J.M., Harder J., Livingston W., Snow M., Woods T. (2011). High-resolution solar spectral irradiance from extreme ultraviolet to far infrared. J. Geophys. Res..

[CR19] Fontenla J.M., Landi E., Snow M., Woods T. (2014). Far- and extreme-UV solar spectral irradiance and radiance from simplified atmospheric physical models. Solar Phys..

[CR20] Garcia R.R. (2010). Atmospheric physics: Solar surprise?. Nature.

[CR21] Haigh J.D., Winning A.R., Toumi R., Harder J.W. (2010). An influence of solar spectral variations on radiative forcing of climate. Nature.

[CR22] Harder J., Lawrence G., Fontenla J., Rottman G., Woods T. (2005). The Spectral Irradiance Monitor: Scientific requirements, instrument design, and operation modes. Solar Phys..

[CR23] Harder J.W., Fontenla J.M., Pilewskie P., Richard E.C., Woods T.N. (2009). Trends in solar spectral irradiance variability in the visible and infrared. Geophys. Res. Lett..

[CR24] Harder J.W., Thuillier G., Richard E.C., Brown S.W., Lykke K.R., Snow M., McClintock W.E., Fontenla J.M., Woods T.N., Pilewskie P. (2010). The SORCE SIM solar spectrum: Comparison with recent observations. Solar Phys..

[CR25] Ineson S., Maycock A., Gray L., Scaife A., Dunstone J., Harder J., Knight J., Lockwood M., Manners J., Woods R. (2015). Regional climate impacts of a possible future grand solar minimum. Nat. Commun..

[CR26] L’Ecuyer T.S., Beaudoing H.K., Rodell M., Olson W., Lin B., Kato S., Clayson C.A., Wood E., Sheffield J., Adler R., Huffman G., Bosilovich M., Gu G., Robertson F., Houser P.R., Chambers D., Famiglietti J.S., Fetzer E., Liu W.T., Gao X., Schlosser C.A., Clark E., Lettenmaier D.P., Hilburn K. (2015). The observed state of the energy budget in the early twenty-first century. J. Climate.

[CR27] Lean J. (2000). Evolution of the Sun’s spectral irradiance since the Maunder Minimum. Geophys. Res. Lett..

[CR28] Lean J.L., Rind D.H. (2008). How natural and anthropogenic influences alter global and regional surface temperatures: 1889 to 2006. Geophys. Res. Lett..

[CR29] Lean J.L., Rottman G., Kyle H.L., Woods T.N., Hickey J.R., Puga L.C. (1997). Detection and parameterization of variations in solar mid- and near-ultraviolet radiation (200 – 400 nm). J. Geophys. Res..

[CR30] Lean J.L., Rottman G., Harder J., Kopp G. (2005). SORCE contributions to new understanding of global change and solar variability. Solar Phys..

[CR31] Mann M.E., Cane M.A., Zebiak S.E., Clement A. (2005). Volcanic and solar forcing of the tropical pacific over the past 1000 years. J. Climate.

[CR32] Marchenko S.V., DeLand M.T. (2014). Solar spectral irradiance changes during cycle 24. Astrophys. J..

[CR33] Matthes K., Funke B., Andersson M.E., Barnard L., Beer J., Charbonneau P., Clilverd M.A., Dudok de Wit T., Haberreiter M., Hendry A., Jackman C.H., Dretzschmar M., Kruschke T., Kunze M., Langematz U., Marsh D.R., Maycock A., Misios S., Rodger C.J., Schaife A.A., Seppälä A., Shangguan M., Sinnhuber M., Tourpali K., Usoskin I., van de Kamp M., Verronen P.T., Versick S. (2016). Solar forcing for CMIP6 (v3.1). Geosci. Model Dev. Discuss..

[CR34] Maycock A.C., Ineson S., Gray L.J., Scaife A.A., Anstey J.A., Lockwood M., Butchart N., Hardiman S.C., Mitchell D.M., Osprey S.M. (2015). Possible impacts of a future grand solar minimum on climate: Stratospheric and global circulation changes. J. Geophys. Res..

[CR35] McClintock W.E., Rottman G.J., Woods T.N. (2005). Solar Stellar Irradiance Comparison Experiment II (SOLSTICE II): Instrument concept and design. Solar Phys..

[CR36] Merkel A.W., Harder J.W., Marsh D.R., Smith A.K., Fontenla J.M., Woods T.N. (2011). The impact of solar spectral irradiance variability on middle atmospheric ozone. Geophys. Res. Lett..

[CR37] Pagaran J., Weber M., Burrows J. (2009). Solar variability from 240 to 1750 nm in terms of faculae brightening and sunspot darkening from SCIAMACHY. Astrophys. J..

[CR38] Pagaran J., Weber M., DeLand M.T., Floyd L.E., Burrows J.P. (2011). Solar spectral irradiance variations in 204 – 1600 nm during the recent Solar Cycles 21 – 23. Solar Phys..

[CR39] Rind D., Shindell D., Perlwitz J., Lerner J., Lonergan P., Lean J., McLinden C. (2004). The relative importance of solar and anthropogenic forcing of climate change between the Maunder Minimum and the present. J. Climate.

[CR40] Rottman G.J. (1988). Observations of solar UV and EUV variability. Adv. Space Res..

[CR41] Rottman G.J., Woods T.N., Sparn T.P. (1993). Solar Stellar Irradiance Comparison Experiment I: 1. Instrument design and operation. J. Geophys. Res..

[CR42] Schlesinger B.M., Cebula R.P. (1992). Solar variation 1979 – 1987 estimated from an empirical model for changes with time in the sensitivity of the solar backscatter ultraviolet instrument. J. Geophys. Res..

[CR43] Schmidtke G., Nikutowski B., Jacobi C., Brunner R., Erhardt C., Knecht S., Scherle J., Schlagenhauf J. (2014). Solar EUV irradiance measurements by the Auto-Calibrating EUV Spectrometers (SolACES) aboard the International Space Station (ISS). Solar Phys..

[CR44] Stephens G.L., Li J., Wild M., Clayson C.A., Loeb N., Kato S., L’Ecuyer T., Stackhouse P.W., Lebsock M., Andrews T. (2012). An update on the Earth’s energy balance in light of new surface energy flux estimates. Nat. Geosci..

[CR45] Swartz W.H., Stolarski R.S., Oman L.D., Fleming E.L., Jackman C.H. (2012). Middle atmosphere response to difference descriptions of the 11-yr solar cycle in spectral irradiance in a chemistry-climate model. Atmos. Chem. Phys..

[CR46] Tapping K.F. (2013). The 10.7 cm solar radio flux (F10.7). Space Weather.

[CR47] Thuillier G., Floyd L., Woods T.N., Cebula R., Hilsenrath E., Hersé M., Labs D. (2004). Solar irradiance reference spectra for two solar active levels. Adv. Space Res..

[CR48] Thuillier G., Foujols T., Bolsée D., Gillotay D., Hersé M., Peetermans W., Decuyper W., Mandel H., Sperfeld P., Pape S., Taubert D.R., Hartmann J. (2009). SOLAR/SOLSPEC: Scientific objectives, instrument performance and its absolute calibration using a blackbody as primary standard source. Solar Phys..

[CR49] Thuillier G., Bolsée D., Schmidtke G., Foujols T., Nikutowski B., Shapiro A.I., Brunner R., Weber M., Erhardt C., Hersé M., Gillotay D., Peetermans W., Decuyper W., Pereira N., Haberreiter M., Mandel H., Schmutz W. (2014). The solar irradiance spectrum at solar activity minimum between Solar Cycles 23 and 24. Solar Phys..

[CR50] Unruh Y.C., Ball W.T., Krivova N.A. (2012). Solar irradiance models and measurements: A comparison in the 220 nm to 240 nm wavelength band. Surv. Geophys..

[CR51] Viereck R.A., Floyd L.E., Crane P.C., Woods T.N., Knapp B.G., Rottman G., Weber M., Puga L.C., DeLand M.T. (2004). A composite Mg II index spanning from 1978 to 2003. Space Weather.

[CR52] Wild M., Folini D., Schär C., Loeb N., Dutton E.G., König-Langlo G. (2013). The global energy balance from a surface perspective. Clim. Dyn..

[CR53] Wild M., Folini D., Hakuba M.Z., Schär C., Seneviraten S.I., Kato S., Rutan D., Ammann C., Wood E.F., König-Langlo G. (2015). The energy balance over land and oceans: An assessment based on direct observations and CMIP5 climate models. Clim. Dyn..

[CR54] Woods T.N., Rottman G.J. (1997). Solar Lyman-alpha irradiance measurements during two solar cycles. J. Geophys. Res..

[CR55] Woods T.N., Pinz D.K., London J., Rottman G.J., Crane P.C., Cebula R.P., Hilsenrath E., Brueckner G.E., Andrews M.D., White O.R., VanHoosier M.E., Floyd L.E., Herring L.C., Knapp B.G., Pankratz C.K., Reiser P.A. (1996). Validation of the UARS solar ultraviolet irradiances: Comparison with the ATLAS 1 and 2 measurements. J. Geophys. Res..

[CR56] Woods T.N., Tobiska W.K., Rottman G.J., Worden J.R. (2000). Improved solar Lyman alpha irradiance modeling from 1947 through 1999 based on UARS observations. J. Geophys. Res..

[CR57] Woods T.N., Eparvier F.G., Bailey S.M., Chamberlin P.C., Lean J., Rottman G.J., Solomon S.C., Tobiska W.K., Woodraska D.L. (2005). The Solar EUV Experiment (SEE): Mission overview and first results. J. Geophys. Res..

[CR58] Woods T.N., Chamberlin P.C., Harder J.W., Hock R.A., Snow M., Eparvier F.G., Fontenla J., McClintock W.E., Richard E.C. (2009). Solar Irradiance Reference Spectra (SIRS) for the 2008 Whole Heliosphere Interval (WHI). Geophys. Res. Lett..

[CR59] Woods T.N., Eparvier F.G., Hock R., Jones A.R., Woodraska D., Judge D., Didkovsky L., Lean J., Mariska J., Warren H., McMullin D., Chamberlin P., Berthiaume G., Bailey S., Fuller-Rowell T., Sojka J., Tobiska W.K., Viereck R. (2012). The EUV Variability Experiment (EVE) on the Solar Dynamics Observatory (SDO): Overview of science objectives, instrument design, data products, and model developments. Solar Phys..

[CR60] Woods T.N., Snow M., Harder J., Chapman G., Cookson A. (2015). A different view of solar spectral irradiance variations: Modeling total energy of six-month intervals. Solar Phys..

[CR61] Yeo K.L., Krivova N.A., Solanki S.K., Glassmeier K.H. (2014). Reconstruction of total and spectral solar irradiance from 1974 to 2013 based on KPVT, SoHO/MDI, and SDO/HMI observations. Astron. Astrophys..

